# A novel *MIP* gene mutation associated with autosomal dominant congenital cataracts in a Chinese family

**DOI:** 10.1186/1471-2350-15-6

**Published:** 2014-01-09

**Authors:** Yibo Yu, Yinhui Yu, Peiqing Chen, Jinyu Li, Yanan Zhu, Yi Zhai, Ke Yao

**Affiliations:** 1 Eye Center, Second Affiliated Hospital of Zhejiang University School of Medicine, No.88 Jiefang Road, Hangzhou, 310009, China; 2 Zhejiang Provincial Key Lab of Ophthalmology, Hangzhou, China

**Keywords:** Autosomal dominant congenital cataract, *MIP*, Y-suture cataract, Nonsense mutation

## Abstract

**Background:**

The major intrinsic protein gene (*MIP*), also known as *MIP26* or *AQP0*, is a member of the water-transporting aquaporin family, which plays a critical role in the maintenance of lifelong lens transparency. To date, several mutations in *MIP* (OMIM 154050) have been linked to hereditary cataracts in humans. However, more pathogenic mutations remain to be identified. In this study, we describe a four-generation Chinese family with a nonsense mutation in *MIP* associated with an autosomal dominant congenital cataract (ADCC), thus expanding the mutational spectrum of this gene.

**Methods:**

A large four-generation Chinese family affected with typical Y-suture cataracts combined with punctuate cortical opacities and 100 ethnically matched controls were recruited. Genomic DNA was extracted from peripheral blood leukocytes to analyze congenital cataract-related candidate genes. Effects of the sequence change on the structure and function of proteins were predicted by bioinformatics analysis.

**Results:**

Direct sequencing of MIP in all affected members revealed a heterozygous nucleotide exchange c.337C>T predicting an arginine to a stop codon exchange (p.R113X). The substitution co-segregated well in all the affected individuals in the family and was not found in unaffected members or in the 100 unrelated healthy controls. Bioinformatics analysis predicted that the mutation affects the secondary structure and function of the MIP protein.

**Conclusions:**

We identified a novel mutation of *MIP* (p.R113X) in a Chinese cataract family. This is the first nonsense mutation of *MIP* identified thus far. This novel mutation is also the first disease-causing mutation located in the loop C domain of MIP. The results add to the list of mutations of the *MIP* linked to cataracts.

## Background

Congenital cataract is the leading cause of visual impairment in children, and it is responsible for approximately 10% of irreversible childhood blindness worldwide, with a prevalence of 1 to 6 cases/10,000 live births [1,2]. It was reported that about 8.3–25% of congenital cataracts are inherited [3], with autosomal dominant transmission the most common mode of inheritance, although autosomal recessive and X-linked traits of inheritance exist [4]. Congenital cataracts may occur in an isolated fashion or in association with other ocular dysmorphology, as well as systemic malformations [3].

Knowledge of the genetic background of congenital cataract has increased considerably during the past decennia. To date, more than 35 independent loci have been identified for nonsyndromic cataract, segregating most often as an autosomal dominant trait, of which 25 represent identified genes [3,5]. The number of mutations exceeds 100 [3,5]. Among the cataract mutations reported, about half involve crystallines, and a quarter involve connexins [3,5,6]. The remainder are divided among the genes for heat shock transcription factor-4 (*HSF4*), major intrinsic protein (*MIP*), and beaded filament structural protein-2 (*BFSP2*) [3,5,6].

*MIP*, a member of the water-transporting aquaporin family, is the most abundant junctional membrane protein in lens fiber cells, constituting more than 60% of the total membrane protein content of these cells [7,8].It plays a critical role in conferring rapid movements of water across cell membranes and controlling the water content of cells [9,10]. To date, several mutations in human *MIP*, including missense and frameshift mutations,have been reported to induce inherited cataracts.

This study aimed to identify the molecular defects in autosomal dominant congenital cataracts in a large Chinese family. And a novel nonsense mutation in *MIP* that co-segregated with the disease was identified to be responsible for the congenital cataracts.

## Methods

### Family enrollment and genomic DNA preparation

A four-generation Chinese family from a remote mountain region of Guizhou province with autosomal dominant congenital cataract (ADCC) was recruited from the Eye Center of the 2nd Affiliated Hospital, Medical College of Zhejiang University, Hangzhou, China. This study was approved by the Zhejiang University Institutional Review Board, and the study protocol followed the principles of the Declaration of Helsinki. After appropriate informed consent was obtained from the participants, all family members underwent detailed ophthalmological examinations, including visual acuity, slit lamp, and fundus examinations with dilated pupils.

Genomic DNA was extracted from the peripheral blood leukocytes using the Simgen Blood DNA mini kit (Simgen, Hangzhou, China) for PCR amplification. A total of 100 ethnically matched subjects withouta family history of congenital cataracts were recruited as controls.

### Mutation screening

We used the functional candidate gene analysis approach. Ten genes most frequently involved in autosomal dominant cataract were analyzed: *CRYAA, CRYAB, CRYBA3/A1, CRYBB1, CRYBB2, CRYGC, CRYGD, GJA3, GJA8,* and *MIP*. All coding exons and intron-exon boundaries of the candidate genes were amplified by PCR using previously published primer sequences [11,12]. The cycling conditions for PCR were as follows: 95°C preactivation for 5 min, 10 cycles of touchdown PCR with 1°C down per cycle from 60°C to 50°C, followed by 25 cycles with denaturation at 95°C for 25 s, annealing at 55°C for 25 s and extension at 72°C for 40s, then finally extension at 72°C for 10 min. The thermal cycling was performed under suitable conditions using a C1000 TM 48-well thermal cycler (Bio-Rad, Hercules, CA). PCR products were isolated by electrophoresis on 1.5% agarose gels and sequenced using the BigDye Terminator Cycle sequencing kit V3.1 (Applied Biosystems, Foster City, CA) on an Applied Biosystems PRISM 3730 Sequence Analyzer, according to the manufacturer’s directions. Sequencing results were analyzed using Chromas 1.62 and compared with sequences from the NCBI GenBank database.

### Bioinformatics analysis

To predict the effect of this nonsense mutation on the protein, we used the online SWISS-MODEL and DEEP VIEW/SWISS-Pdb tool to analyze both the mutant and wild-type version of the structure of the MIP protein. For hydropathy analysis, we used Compute pI/MW to predict the isoelectric point (pI) and the molecular weight (MW) of the wild-type and mutant protein. Furthermore, online Mutation Taster software was used to distinguish between functionally neutral and deleterious mutations.

## Results

### Clinical evaluation

**Figure 1:**
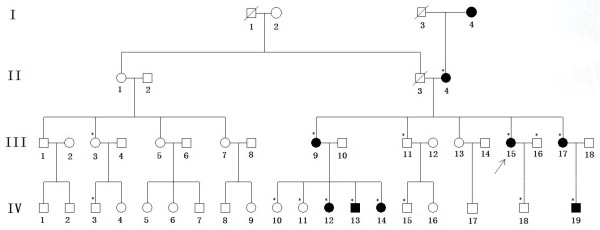
**Pedigree of the four-generation Chinese family with ADCC.** Squares and circles indicate males and females, respectively. Black symbols indicate affected members and open symbols indicate unaffected individuals. The diagonal line indicates a deceased family member and the black arrow indicates the proband. Asterisks indicate family members who attend this study.

**Figure 2:**
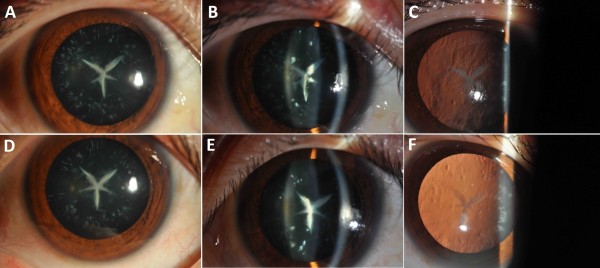
Slit lamp photographs (diffuse illumination, silt lamp and retro-illumination) of the proband (III:15) which shows bilateral Y-suture cataract combined with punctuate cortical opacities, A, B, C denotes the right eye and D, E, F denotes the left eye, respectively.

We identified a four-generation Chinese family (8 affected and 8 unaffected) with a clear diagnosis of ADCC (Figure 1). The proband was a 26-year-old female who had underwent bilateral cataract surgery in our hospital in March and May 2013 to obtain a driving license. To obtain more information about the cataract phenotype and to collect the blood samples, we went to their place of residence and identified the identical phenotype within all affected members. The phenotype was a typical bilateral Y-sutural cataract, combined with punctuate cortical opacities (Figure 2). The older affected members (II:4,III:9,III:17) were aged from 23 to 55 years and the younger ones (IV:12,IV:13,IV:14,IV:19) were aged from 3 to 10 years. None of the affected patients whose visual acuity ranged from 0.6 to 1.0 complained of significant visual deterioration, and they were also unaware of their cataracts until the examinations. As they had not underwent ocular examinations before, we were unable to determine the exact age of onset of the cataract. However, we may speculate that all the affected individuals showed early onset of cataract because the youngest member (IV:19) already had a mild cataract. Other than the cataracts, there were no other ocular or systemic abnormalities or symptoms.

### Mutation screening

**Figure 3:**
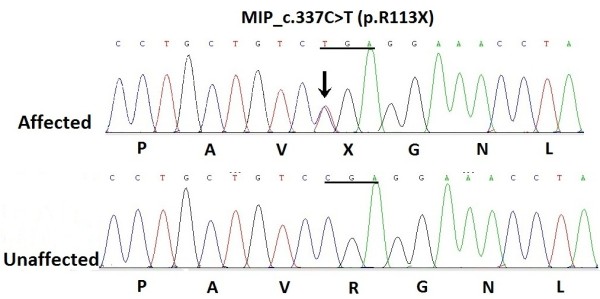
**DNA sequence chromatograms of the proband (III:15) shows a heterozygous C > T nucleotide change in exon 1 of *MIP *** **(black zrrow) which altered the Arg to a stop codon (TGA) and an unaffected individual (III:12) shows CGA at the same codon 113.**

We identified a heterozygous change, C > T, at position 337 (c. 337C > T) of the *MIP* gene, leading to the replacement of a wild-type arginine with a stop codon at the 113th amino acid position (p.R113X) (Figure 3). It co-segregated well with all affected individuals and was not found in unaffected family members or in the 100 unrelated normal controls. This strongly suggests that the R113X mutation may act as a disease-causing mutation rather than a benign polymorphism in linkage disequilibrium with the disease.

### Bioinformatics analysis

**Figure 4:**
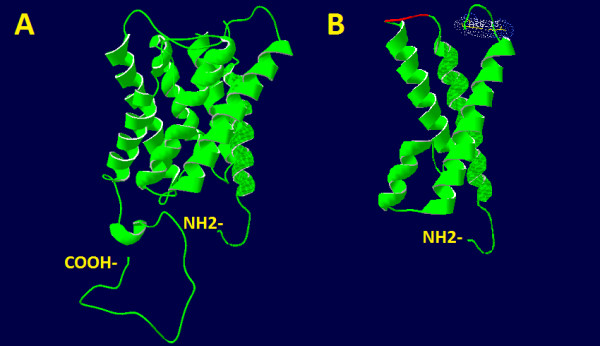
**The structural model of the *MIP *protein. A**: A structural homology model of the wild-type human *MIP* protein is displayed. **B**: A structural alteration of the mutant *MIP* is displayed. 151 amino acids are truncated from the COOH-terminus of *MIP* as a result of c.337C > T mutation.

The automated homology protein of human MIP was modeled in three dimensions by the Swiss-Model and DEEP VIEW/SWISS-Pdb tool. The results clearly showed that the shortened protein contained only 113 amino acids rather than the normal 263 (Figure 4). This gave rise to a severely truncated protein that lacks important functional domains on the C-terminal region. The theoretical pI of mutant MIP was increased to 9.18 compared to the wild-type pI of 8.62. The MW of the mutant (11956 Da) was significantly reduced compared to the MW of the wild-type MIP (28121 Da), as predicted by Compute pI/MW. Results obtained with the online bioinformatics software Mutation Taster showed that the mutation was predicted to be “probably damaging.”

## Discussion

As the most abundant membrane protein within lens fiber cells, *MIP* facilitates the movement of water into and across lens fiber cells. Besides functioning as a water channel, it may also act as an adhesion molecule, compacting highly ordered fiber cells and minimizing extracellular space and light scattering to maintain the lens transparency [13]. To date, 10 mutations of *MIP* have been associated with congenital cataract (c.97C > T [p.R33C] [12], c.401A > G [p.E134G] [14], c.413C > G [p.T138R] [14], c.530A > G [p.Y177C] [15], c.559C > T [p.R187C] [16], c.698G > A [p.R233K] [17], c.2 T > C [p.Met1?] [18], c.494G > A [p.G165D] [19], IVS-1G > A [p.V203fs] [20], and c.638delG [p.G213VfsX46] [21]). As we all know, the dominantly inherited mutations are mainly missense mutations that lead to amino acid substitutions, other examples include nonsense or frame shift mutations. And the severity of the cataract may be determined by anatomic location, size, density, and progression of the opacity. In general, the more posteriorly located and dense an opacity, the greater the impact on visual function [2,22]. The cataract family we reported was associated with a nonsense mutation (c.337C > T [p. R113X]) which produced a severely truncated protein. While this mutation produced an identical phenotype of Y suture cataract combined with fine punctate opacities in the cortex which did not involve the posterior part of the lens. This would well explain why there was no complaint of distinctly decreased visual acuity from all patients.

The phenotypes of cataracts are significantly different among *MIP* mutation families, pointing to the presence of extensive clinical heterogeneity of hereditary cataracts. We compared the phenotype of our family with one affected member of a cataract family reported by Geyer et al. [21] in 2006 and found that the clinical features were very similar: Both manifested as fine punctate opacities in the cortex and Y suture. They reported a single nucleotide deletion, which caused a frameshift and premature stop codon that truncated 6 amino acids from the C-terminus of MIP. More interestingly, when we analyzed the phenotypes of other reported families, we found that 3 of 10 examples (30%) had opacities involving Y sutures [14,18,21]. The way in which the lens suture forms may explain this finding. As is well known, *MIP* is expressed as soon as the first primary fibers start filling the lens vesicle, and it continues to be expressed as the secondary fibers are differentiated from the equatorial epithelial cells [23]. When the terminal ends of the secondary fibers overlap with one another, lens sutures form [24]. Deletion of the *MIP* gene in mice led to a lack of suture formation [25,26]. The important role that *MIP* plays in suture formation is consistent with the phenotype observed in *MIP* mutation families.

**Figure 5:**
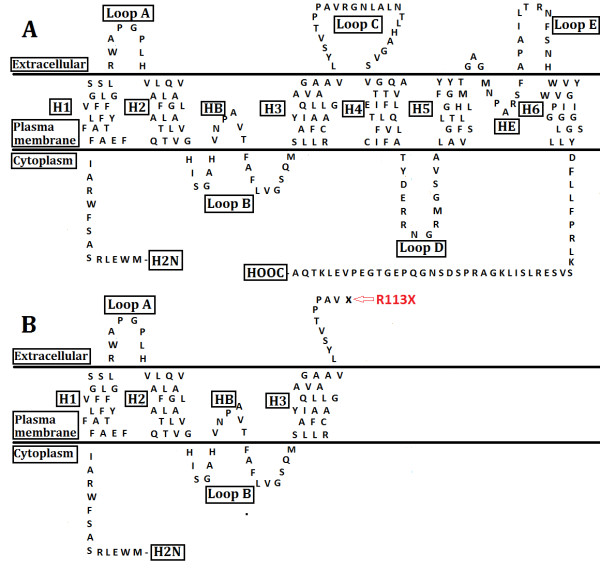
**The schematic diagram of the protein. A**: The schematic diagram of the predicted structure of *MIP* protein (modified from Watanabe K et al. [28]) encoded by the wild-type. **B**: The mutated schematic illustrates premature truncation of the protein. The locations of six transmembrane helices (H1, H2, H3, H4, H5, and H6), two hemichannels (HB and HE), the extracellular (Loop A, C, and E) and intracellular (Loop B and D) loops and two asparagine–proline–alanine (NPA) motif are indicated.

Four mutations, E134G [14], T138R [14], G165 D [19], and c.638delG [21], have been functionally characterized in vitro. The E134G, T138R [23], and G165D mutations may result in loss of water permeability due to failure in trafficking of proteins to the plasma membrane. The 638G deletion [27] may cause the mutant protein to be retained in the endoplasmic reticulum and induce cellular cytotoxicity. The nonsense mutation identified in this study encodes a premature stop codon, resulting in a shortened protein with 151 amino acids. This produces a severely truncated protein and deletes important functional domains on the C-terminal domain, including loop C, D, E, transmembrane domains (H4-H6), and hemipores HE (Figure 5). The shortened protein may alter voltage dependence and calmodulin-binding properties of the protein. Our finding is quite similar to the *Mip* mutation identified in KFRS4/Kyo cataract rats by Watanabe et al. [28]. In their study, they found a 5-bp insertion in the *Mip* gene that generated a frameshift and a premature stop codon at amino acid position 127, which produced a truncated protein that lacks 136 amino acids in the C-terminal region. No MIP-positive band in the eye extracts from the mutant rats was detectable by immune-blot, and a lack of MIP-specific immune-fluorescence in eye sections from the mutants also indicated the absence of normal protein. These results suggest that the *kfrs4* mutation conveys a loss-of-function, which leads to functional inactivation though the degradation of *Mip* mRNA by nonsense-mediated mRNA decay (NMD). The NMD pathway is an effective mRNA surveillance system that detects and degrades mRNA containing premature termination codons (PTC) and protects cells from potentially deleterious effects of truncated proteins [29-32]. As the site and resultant truncation of the mutation identified in the present study are quite similar to the KFRS4 rat mutation, we may speculate that the mutant transcripts of this nonsense mutation may escape the NMD pathway and that PTC-mRNAs are translated to abnormal proteins, with potential dominant-negative effects on cells. Further study is needed to provide insights into the molecular consequence of this nonsense mutation.

## Conclusion

In this study, we have described the first nonsense mutation in *MIP* causing autosomal dominant congenital cataracts in a large Chinese family. It is also the first mutation located in the loop C domain of MIP. Our data expand the spectrum of *MIP* mutations and validate the extensive clinical and genetic heterogeneity of congenital cataract. None of the affected members in the family complained of significant visual deterioration in their daily life. The identification of this mutation may enable proper genetic diagnostics and counseling in both young and elderly patients.

## Competing interests

The authors declare that they have no competing interest.

## Authors’ contributions

YY and KY obtained the funding, designed the research and revised the manuscript. YY carried out the molecular genetic studies, participated in the sequence alignment, analyzed most of the data, and drafted the manuscript. JL and YZ carried out sample collection and helped prepare the laboratory work. PC and YZ participated in the design of the research, help the acquisition of the clinical data and revision of the manuscript. All authors have read and approved the final manuscript.

## Pre-publication history

The pre-publication history for this paper can be accessed here:

http://www.biomedcentral.com/1471-2350/15/6/prepub
